# Using a SPATIAL INS/GNSS MEMS Unit to Detect Local Gravity Variations in Static and Mobile Experiments: First Results

**DOI:** 10.3390/s23167060

**Published:** 2023-08-09

**Authors:** Benjamin Beirens, José Darrozes, Guillaume Ramillien, Lucia Seoane, Patrice Médina, Pierre Durand

**Affiliations:** 1 Université Toulouse 3—Paul Sabatier, 115C Route de Narbonne, 31062 Toulouse, France; bbeirens@hotmail.com (B.B.); lucia.seoane@get.omp.eu (L.S.); 2 Géosciences Environnement Toulouse (GET), Observatoire Midi-Pyrénées (OMP), 14, Avenue Edouard Belin, 31400 Toulouse, France; guillaume.ramillien@get.omp.eu; 3 Centre National de la Recherche Scientifique (CNRS), 3 Rue Michel Ange, 75016 Paris, France; patrice.medina@aero.obs-mip.fr (P.M.); pierre.durand@aero.obs-mip.fr (P.D.); 4 LAERO-Laboratoire d’Aérologie, Université de Toulouse, Centre National de la Recherche Scientifique, 31400 Toulouse, France

**Keywords:** MEMS INS/GNSS, gravimetry, solid tides, airborne

## Abstract

In this study, we present the feasibility of using gravity measurements made with a small inertial navigation system (INS) during in situ experiments, and also mounted on an unmanned aerial vehicle (UAV), to recover local gravity field variations. The INS operated is the SPATIAL one developed by Advanced Navigation, which has three-axis accelerometers. When the temperature bias is corrected, these types of INS are powerful enough to present the periodic signal corresponding to the solid Earth tides. There is also a clear correlation with the data measured at different altitudes by a CG5 gravimeter. However, these data were recorded on static points, so we also studied the INS in a moving platform on a UAV. Because there are a lot of vibrations recorded by the INS (wind, motor, on-board computer), the GPS and accelerometric data need to be filtered extensively. Once the data are corrected so they do not show thermal bias and low-pass filtered, we take the second derivative of the altitude (GPS) data to find the radial accelerometry of the drone and compare it to the radial accelerometry measured directly by the INS, in order to isolate the accelerometric signal that is related to the area that is being studied and the altitude. With a high enough precision, this method could be used to obtain the gravity variations due to the topography and density variations in the ground.

## 1. Introduction

Studies have been conducted before using INS and GPS measurements mounted on vehicles in order to obtain gravity data covering a large area. The instruments used in previous studies, however, were large inertial measurement units such as the iNAV-RQH [1,2], and because of their size and weight, the studies were limited to cars, vans, planes or quadricopter drones. We used a small INS, with the size and weight being close to a USB flash drive so that it can easily equip on a wing drone, which can cover an area much quicker. The INS used was the SPATIAL model developed by Advanced Navigation. This instrument is able to measure acceleration, velocity, magnetic field along three axes, the pitch, roll and yaw, temperature and pressure all at a very high frequency rate (up to 1000 Hz). This type of instrument, as with a larger INS, is made for accelerometric measurements and not for gravity purposes, but with gravity being considered as an acceleration, we wanted to figure out if the system is precise enough to be used for gravity applications and not just high-precision position determination through the combination of GNSS/INS. The goal is to use this system for airborne gravimetry to have a completing view of gravimetry all the way from the spaceborne gravity field data from satellite missions (CHAMP, GRACE, GOCE [3,4]) down to local or regional terrestrial data obtained with gravimeters. We first present the study of the behavior of this instrument in a static environment at the GET (Géosciences Environnement Toulouse) laboratory, in order to study the influence of various factors on its precision, most notably temperature. After this we compare the data measured over the course of multiple weeks with that of the theoretical signal for the solid Earth tides [5]. After the correction of the temperature bias, we observed a wavelet coherence between the INS data and the periodic signals of the solid Earth tides given the position and time of our experiment. Then we attached the INS to the inside of a van during a round trip to the Cammazes dam, which has an altitude difference of 400 m with the GET laboratory. We compare the measurements with that of a precise CG-5 gravimeter. Finally we present the data of the MEMS INS/GNSS when mounted on a wing drone during a 2 h flight. The accelerometric signal recorded by the accelerometers was reduced as much as possible through low-pass filtering because an important amount of parasitic noise was recorded due to vibrations (wind, motor, on-board computer).

## 2. State of the Art

Mobile gravimetry is not a new concept; for years now, new methods have been developed and tested that combine gravimeter and GPS measurements in order to fill the gap of scale between spaceborne gravity data and that of local networks.

In 2001, C. Jekeli wrote a definitive textbook on geodetic applications and inertial navigation. One of his research focus points was INS/GPS airborne vector gravimetry, and one of his publications “New approach for airborne vector gravimetry using GPS/INS” [6,7], is very similar to our approach. However, the system they are developing can only be used on airborne platforms carrying very large payloads, which is very different from the approach we are developing, which is dedicated to UAVs with very small payloads (<10 kg). Kinematic accelerations of an INS were used as updates, rather than velocities and positions. Data from a test flight were also used and the calculations were performed in the inertial frame. A precision as low as 3–4 mGal along the vertical axis was obtained, with a precision of 6 mGal along the horizontal axis. In 2008, the National University of Defense Technology (NUDT) in China made the SGA-WZ, the first Chinese airborne gravimetry system that was developed from a strapdown INS (SINS) combined with a differential GNSS (DGNSS) system [8]. In 2013, Cai et al. ([9]) published their results using this system on a Cessna 208 plane. During the test flight, the plane followed the same lines multiple times over the course of six flights; this allowed them to test the repeatability. The length of the repeated lines was 100 km and the plane had a relatively consistent speed of 216 km/h. Using a filter period of 160 s, they obtained a spatial resolution of 4.8 km with a precision as small as 3.2 mGal for their repeated lines (with 1 mGal = $10^{-5}$ m/s${}^{2}$). Their method was inspired by other airborne gravimetry studies using a SINS/GNSS [10,11].

In 2019, researchers in China developed a small gravimeter based on microelectromechanical systems (MEMS) technology with a similar sensitivity to that of a large gravimeter. Their instrument was tested alongside a GWR iGrav superconducting gravimeter to measure the effects of solid Earth tides and obtained a correlation coefficient of 0.91 [12].

Three years later, a similar study of the solid Earth tides was conducted with a MEMS gravimeter, but this time, a correlation of 97.5% was obtained. The sensors for MEMS gravimeters are cheaper to produce than large gravimeters because they rely on the same fabrication techniques used to produce accelerometers [13].

An autonomous mobile gravimetry system, “LIMOG” (LIght MOving Gravimetry), was developed [14]. This system combined three “QA 3000-020” accelerometers and multiple GPS antennas to measure the accelerometry along three axes. These accelerometers had already been used on satellites, planes and even missiles. The tetrahedron system created was mounted on a van or boat as it also had to be connected to a receptor and battery inside the vehicle. This latter system was proven to be precise enough to have a milligal precision.

Recently, in 2022, a team of French researchers improved the LIMOG system and developed an instrument, called ”GraviMob”, for the measurement of underwater gravity anomalies. The system consists of triads of accelerometers attached to an autonomous underwater vehicle (AUV) [15].

An INS/GNSS UAV-borne vector gravimetry system was developed [1]; the INS used was an iNAV-RQH, which weighs 9 kg, and because of this, it was installed on an unmanned helicopter. In kinematic mode, this system has a vertical precision of 4 mGal.

Most recently, a group of Chinese researchers published an article [16] on the integration of an SGA-WZ04 gravimeter on a CH-4 UAV (developed by the National University of Defense Technology, NUDT). They surveyed the same line many times by repeatedly flying over it to accumulate data and compared them with those of EGM2008. This study was inspired by the previous study of 2013 [9] and their follow-up work [17,18].

While these systems proved to be precise enough for mobile gravimetry, the size, weight and cost are still very limiting. Of the studies mentioned, the most comparable one is [16]. The main difference and the aim of our study compared to theirs, is that we used a much smaller (4 × 3 × 2.5 cm), low-cost instrument (MEMS INS/GNSS vs gravimeter), which barely added any weight when mounted on a vehicle, such as a small wing drone. The drone we were able to use was the Boreal LAB model. With a wingspan of 3 m and a maximum weight of 25 kg, the advantage of this type of drone is that it can be ready for launch in as little as 30 min and is able to cover hard-to-reach areas much quicker [19,20], but the disadvantage is that less precise measurements can be obtained as the SPATIAL system is affected much more by noise than larger INSs or gravimeters. We also tested this system using in situ experiments and not just mobile experiments.

## 3. Materials and Model

### 3.1. Inertial Navigation System—SPATIAL

**Figure 1:**
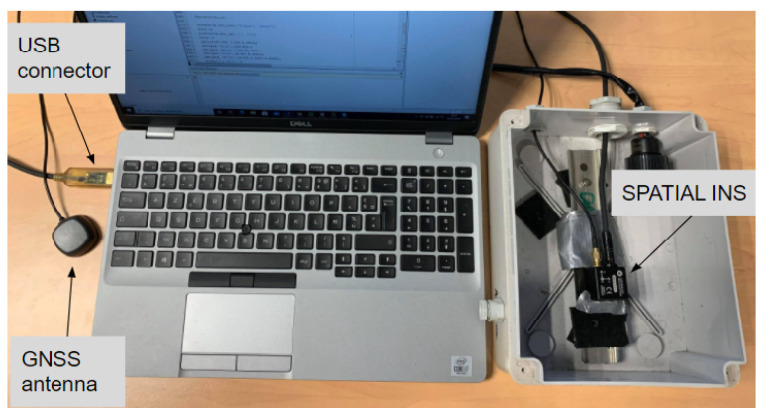
SPATIAL inertial navigation system connected to a computer through USB and a GNSS antenna.

As mentioned before, the inertial navigation system used is the miniature GNSS-aided INS SPATIAL model, which can be seen on Figure 1, designed by Advanced Navigation. It has, in real-time kinematic (RTK) mode, a horizontal and vertical precision of 0.03 and 0.02 m, respectively, when used with an L1 RTK antenna (see Table 1). It has a bias instability of 2.$10^{-4}$ m/s² (see Table 2) but also has a not clearly defined temperature sensitivity bias, known as $g_{d}$ the zero drift (see Equation (1)). Our aim is to study if these miniature INSs/GNSSs are precise enough not only for the gravity measurements and separate different gravity contributions, such as altitude variations, solid Earth tides and the topography of an area, but also local variations in the hydrology (surface water, moisture, ground water).

### 3.2. Reference—CG-5 Autograv Gravimeter

The sensor of the CG5 gravimeter is a fused quartz spring system. The CG-5 gravimeter has a standard resolution of 1 μGal ± σ (see Table 3). The gravitational force on the proof mass is balanced by a spring that is associated with an electrostatic restoring force. This system that is associated with a durable shock mount system permits the instrument to be operated without clamping.

### 3.3. Drone—Boreal LAB

The drone used is a long-range civil unmanned aerial vehicle (UAV) of the fixed-wing single rotor drone type (manufactured by BOREAL SAS). The model is the Boreal LAB, designed specifically for scientific survey missions. It can carry a payload of up to 7 kg and is specialized for reconnaissance missions. A 60 W generator is integrated to provide continuous power to the scientific instruments. This UAV can fly for up to 8 h, has a minimum speed of 70 km/h and a maximum speed of 130 km/h. This type of drone can be launched from almost any type of terrain and can be deployed in as little as 30 min. The INS is attached inside the drone so that its *x*-axis points towards the front of the drone, the *y*-axis points towards its right wing and the *z*-axis is pointing down. The MEMS INS is attached as close as possible to the center of gravity of the UAV, so that the inertial reference frame and the UAV reference frame are assumed to coincide.

### 3.4. Earth Tide Model Used

In our study, to validate the results obtained by the MEMS INS, we decided to compare our measurements with the well-known theoretical solid tide model called Tsoft [21]. This software predicts the periodical gravity variations due to solid Earth tides. It integrates preprocessing to correct the artifacts of the analyzed time series (outlier, step, missing data). For this purpose, it is based on powerful filtering tools either in the frequency domain (FFT) or in the time domain (e.g., 2-poles Butterworth filter).

Land tides or solid tides are also defined as body tides in solid Earth geosciences [22]. As with ocean tides, the Moon has the greatest effect on body tides because it is closer to the Earth. The Sun does have an effect on body tides as well because of its very large mass and its strong gravitational field. As the Earth rotates around the Sun and the Moon, each of their gravitational fields pull on the Earth. Because of this pull, there are small deformations (or bulges) on the Earth’s surface known as body tides. These bulges face the Moon and the Sun as the Earth rotates. The Tsoft model predicts the Earth’s displacement and gravity change due to Earth tides in time but also in space. Body tides are lower than ocean tides, and the displacement of the Earth’s surface is usually no greater than ±30 cm. A simplified model is given to express the gravity solid tides, $g_{s}$, [22]:

(1) $$\begin{array}{c} g_{s}=g_{0}-g_{d}-g_{a}-g_{oc}-\epsilon \end{array}$$

where $g_{0}$ are gravity observations, $g_{d}$ the zero drift (for spring gravimeter and for INS due to temperature), $g_{a}$ the atmospheric load, $g_{oc}$ the ocean loading and ϵ represents errors and noises. Removing $g_{0}$, $g_{d}$, $g_{a}$, $g_{oc}$ and then $g_{s}$ is regarded as the sum of different solid tide waves and can be decomposed in harmonic terms, i.e., simple periodic Fourier series according to [22]:

(2) $$\begin{array}{c} g_{s}=\sum_{m=1}^{k}\delta_{m}\sum_{n=m_{\alpha }}^{m_{\beta }}W_{mn}\cos\left(\omega_{mn}t+\varphi_{mn}+\Delta \varphi_{m}\right)+\varepsilon \end{array}$$

where *k* corresponds to the number of wave groups (WG), $m_{\alpha }$ and $m_{\beta }$ are the respective number of beginning and ending tide waves in the $m^{th}$ WG, $\delta_{m}$ and $\Delta \varphi_{m}$ are the corresponding amplitude factor and phase delay of the WG, and $W_{nm}$, $\omega_{nm}$ and $\varphi_{nm}$ are the theoretical amplitude, frequency and initial phase of *(n,m)* tide waves, respectively. Finally, according to the least square solution, the δ and Δφ of all tide waves and their associated Root-Mean-Square (RMS) errors are determined, and the gravity solid tidal model is computed.

## 4. Static, Mobile and Airborne Mission

### 4.1. Solid Earth Tides (Static Mode)

#### 4.1.1. Methodology of In Situ Experiment at the GET Laboratory

Before using this INS for any gravity studies, we need to study the behavior of this instrument depending on different variables such as sampling frequency, temperature and pressure. At the same time, we must also determine if this system can detect the low-magnitude gravity response due to the solid Earth tides as this will indicate if the INS has a good enough precision for other applications. These in situ studies were performed at the GET laboratory (Toulouse, France) over a two-month period during the summer of 2020, and the records were compared to the ones of a CG-5 Autograv gravimeter considered as a reference. The absolute value of the vertical acceleration, measured at this location with an absolute gravimeter with a precision of $10^{-8}$ m/s², is 9.80462552 m/s² (980,462.552 mGal).

**Figure 2:**
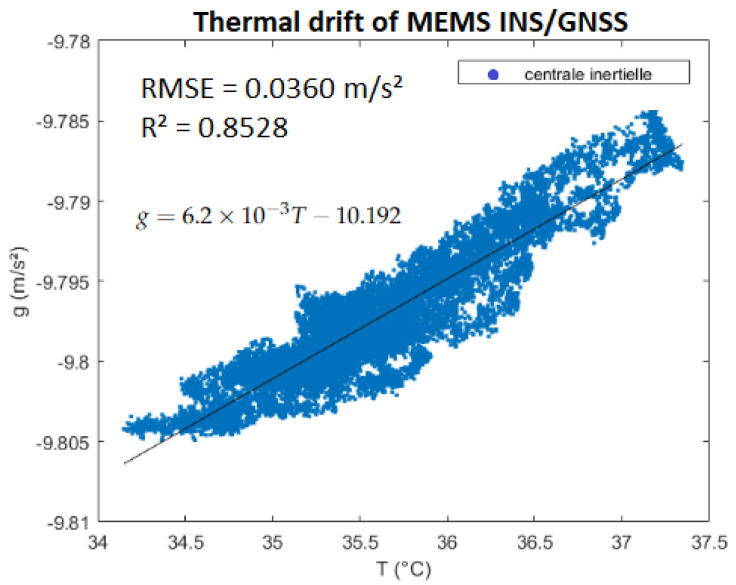
MEMS INS/GNSS thermal drift, g represents the gravity uncorrected for thermal drift (18,720 points).

Static measurements in the laboratory show us that the MEMS INS/GNSS has a clear temperature dependence. As we can see in Figure 2, which plots the raw vertical acceleration data along the z-axis of the INS/GNSS versus the recorded temperature, the thermal drift follows a linear law in this temperature range, which means it can easily be corrected empirically. Because of a high noise density, the experiment is conducted at a lower sampling rate (0.0167 Hz, 1 measurement each minute) as the solid Earth tides have much larger fundamental periods.

**Figure 3:**
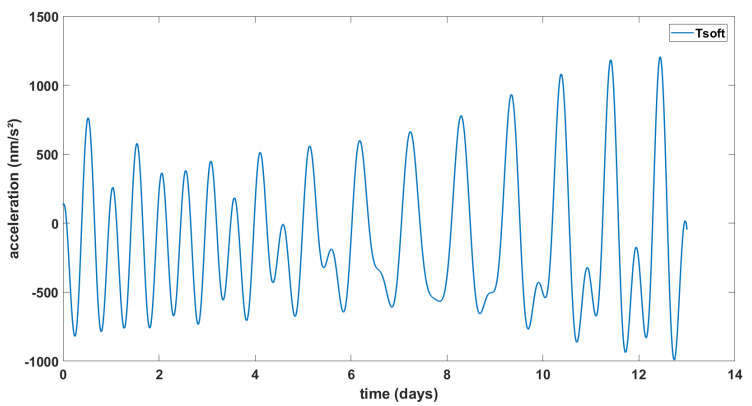
Theoretical solid Earth tide signal (Tsoft) calculated for the gravity station located at the GET laboratory for the period of 3–15 September 2020.

We obtained the vertical gravity signals from the solid Earth tides using the Tsoft software, for the period and geographical coordinates of our recorded data, as seen in Figure 3. On the 3rd of September 2020, the Moon is 98% illuminated (1 day after the Full Moon), which is shown by the semidiurnal solid tides. On the opposite side, on the 15th of September, the Moon is 5% illuminated, 2 days before the New Moon. There, we see the same semidiurnal cycle appearing and the amplitude is higher for both of these periods because the gravitational pull of both the Moon and the Sun are working together. For the middle part of this period, the Sun’s and the Moon’s positions are at, or close to, a 90° angle. Their gravitational pulls counteract the effects of each others, which is why the amplitude is lowest for this period and the semidiurnal cycle disappears.

Using the data recorded by the MEMS INS/GNSS and the theoretical data from Tsoft, we proceeded by applying the temperature correction to the raw data before computing the continuous wavelet transform (CWT) of both signals. With the CWT of each signal, the cross wavelet transform (XWT) was computed, which highlights the correlation in time for different periods between two signals, even when they are not in phase. By decomposing the signals into their frequency domains and applying a low-pass filter (sub 10 h), we were able to get rid of the noise of the INS/GNSS, and then we reconstructed the signal with the remaining frequencies.

**Figure 4:**
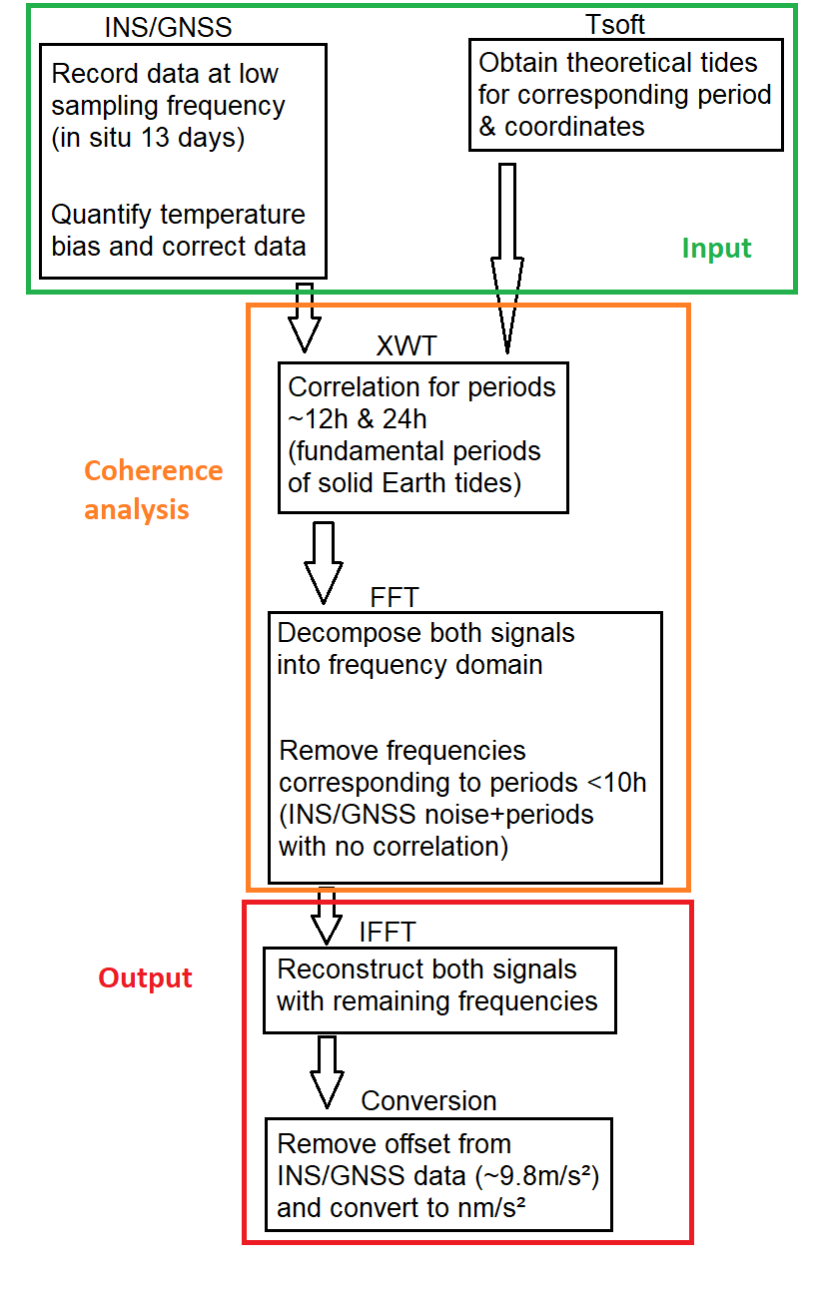
Flow chart of the different steps performed to treat the INS/GNSS and Tsoft data.

A schematic representation of the steps of the processing chain is presented in Figure 4.

#### 4.1.2. Results of In Situ Experiment

**Figure 5:**
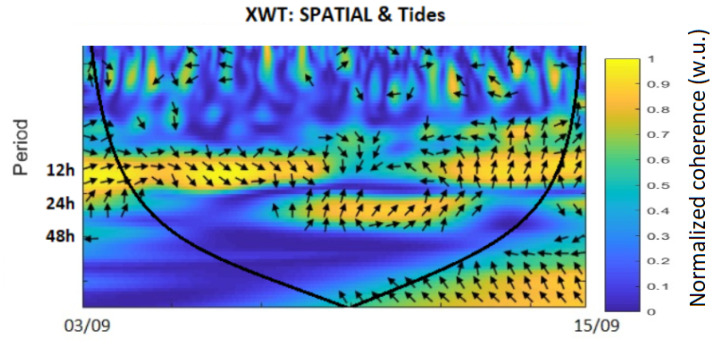
Wavelet coherence between theoretical solid Earth tides (Tsoft) and accelerometric INS data.

**Figure 6:**
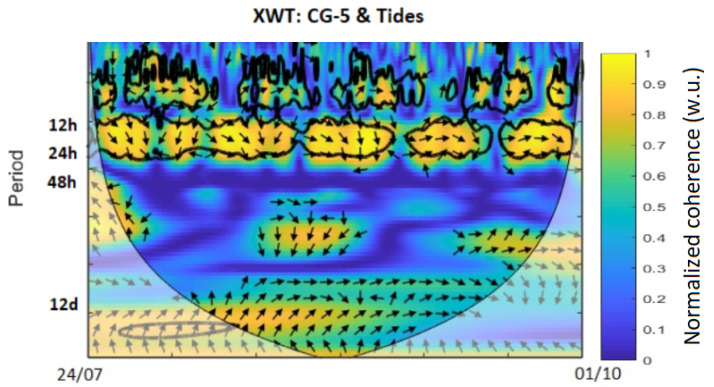
Wavelet coherence of theoretical solid Earth tides (Tsoft) and CG-5 data.

In order to characterize the correlations that exist between the theoretical solid tide model and the measurements made by the INS (Figure 5) and those made with the CG5 (Figure 6), we carried out a wavelet cross-correlation [23], which is a tool for deconvoluting and comparing signals on different scales [24]). When the raw INS data are corrected for the temperature bias $g_{d}$ (Figure 2), and the wavelet coherence of the theoretical solid Earth tides and the accelerometric MEMS INS/GNSS data are compared for that period, a strong correlation between the daily and sub-daily periods of 12 to 24 h is noticed in Figure 5. This same coherence is found between the theoretical tides and the reference data from the CG-5 Autograv gravimeter in Figure 6. The CG-5 gravimeter recorded uninterruptedly for two months and showed a correlation for a period of 13 days. The upper part of these figures shows no correlation, which is to be expected since we would only notice the noise of INS is present for such low periods and the theoretical tide data obtained from Tsoft do not contain any noise. Any correlations outside the cone of influence, represented by the black/grey line in Figure 5 and Figure 6, are ignored as these are due to edge effects.

**Figure 7:**
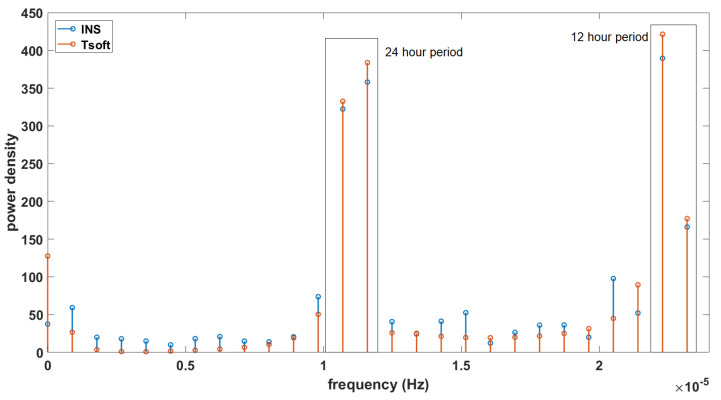
Frequencies corresponding to the periods of 12 h and above of the Tsoft and INS data.

**Figure 8:**
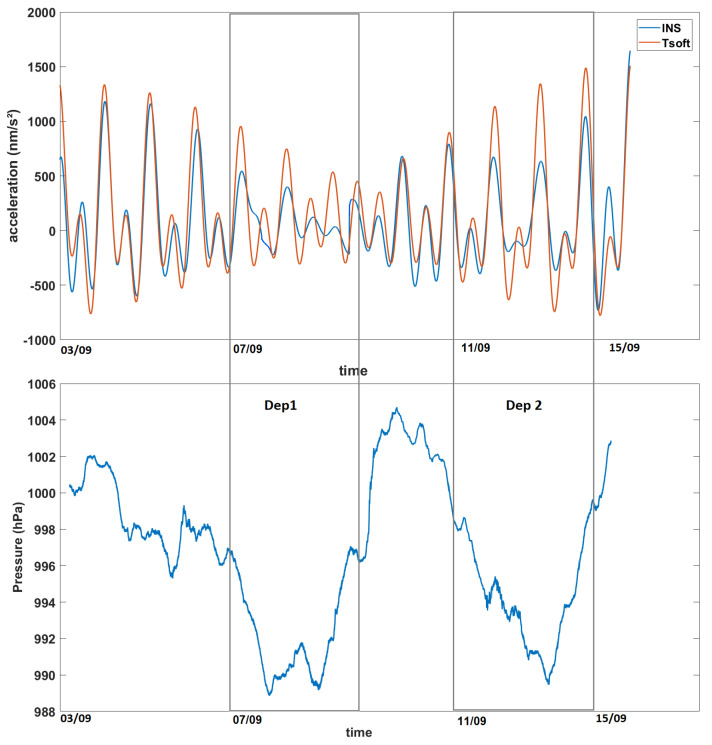
Reconstructed signals (theoretical and INS/GNSS data) after removing the frequencies corresponding to the periods < 10 h; below shows the atmospheric pressure recorded by the INS/GNSS for the same period (Dep1 and Dep2: lower atmospheric pressure).

By decomposing the Tsoft and INS signals in their frequency domain in Figure 7, we can see that both of these signals have important amplitudes for the frequencies corresponding to the periods of 12 and 24 h (which are also the periods with a high coherence on the cross wavelet transform). If all the periods are filtered out for 10 h and below (as there are no frequencies with a high correlation for periods under 12 h) and we reconstruct the signals from the remaining frequencies, we obtain the residuals presented in Figure 8. These residuals of the MEMS INS/GNSS were converted into nm/s² (the units in which the Tsoft signal is given) and an offset of ∼9.8 m/s² was removed so that the local gravity anomalies and not the absolute values were obtained. The correlation between these signals is ∼0.84 and the RMSE 405 (nm/s²). The biggest amplitude difference between the residual signals can be seen near the middle part (4–5 days after the start of the experiment) and at the end (2–3 days before the end of the experiment). Change in atmospheric pressure can cause this difference from the theoretical tide. The atmospheric pressure recorded by the INS/GNSS is presented in Figure 8 (bottom), where significant changes in the middle (07/09) and at the end (11/09) of the period are seen, the same periods where a change in the gravity residuals’ amplitude can be noticed.

### 4.2. Altitude Variations (Mobile)

#### 4.2.1. Methodology of Cammazes Dam Excursion

**Figure 9:**
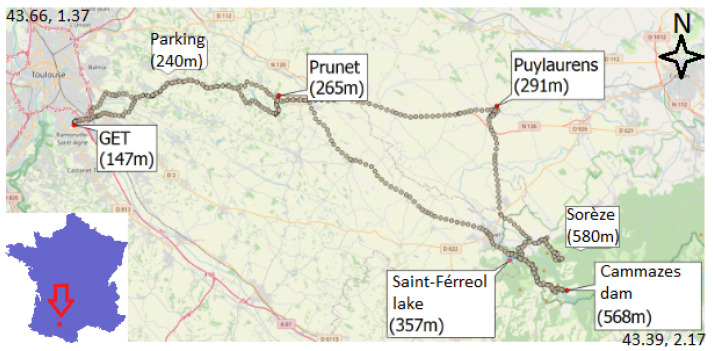
Route taken from the laboratory to the Cammazes dam and the corresponding altitudes at each stop written in black.

Before installing the MEMS INS/GNSS in a drone, we placed the INS in a moving ground vehicle. The INS was installed inside a van and we drove to the Cammazes dam (Tarn, France). The goal was to see if the INS would be sensitive to the gravity variations due to the 400 m altitude difference between the GET laboratory and the Cammazes dam. On the way, eight stops were made in order to obtain measurements from the CG-5 gravimeter as well, as they were used as a reference for the MEMS INS/GNSS measurements. The trajectory throughout the day can be seen in Figure 9.

The first measurements of the MEMS INS/GNSS at the GET laboratory were calibrated to the measurement from the CG-5 gravimeter. The CG-5 gravimeter itself was calibrated with the absolute value at Puylaurens, which is an Institut Géographique National (IGN) reference point. This mission lasted 10 h and the sampling frequency was set to 0.033 Hz (or 1 measurement/30 s); thus, between the start of the journey and final arrival, the INS experienced significant temperature variations, so the linear law was used from Figure 2 as the MEMS INS/GNSS instrument was the same one. Unlike the in situ experiment at the laboratory, during this excursion, the INS/GNSS would not have been positioned perfectly horizontal at each location. Because of this, a rotation matrix was applied to its acceleration data, using the pitch, roll and yaw from the gyroscopes, measured by the MEMS INS/GNSS for correction of the data, to ensure they were in the NED-frame. The MEMS INS/GNSS itself was attached as close as possible to the center of gravity of the vehicle so that both reference frames would coincide.

#### 4.2.2. Results of Mobile Experiment

**Figure 10:**
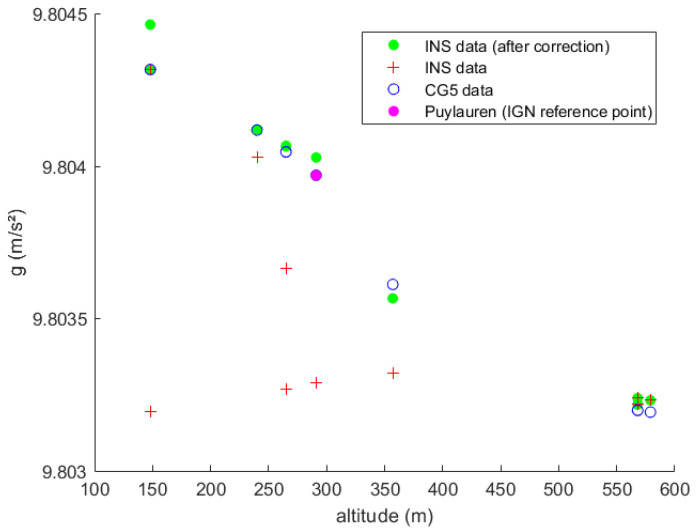
Gravity measurements with CG5 Autograv gravimeter and SPATIAL INS during Cammazes mission.

At each stop location, we recorded using the CG-5 gravimeter and INS/GNSS, and the data were compared to validate our gravity measurements. In Figure 10, the red crosses represent the MEMS INS/GNSS data at each stop location before temperature correction and the green dots represent the gravity data after temperature correction. The blue circles show the data recorded by the CG5 gravimeter (after correcting the drift), which are used as the reference gravity values. Because this mission was a round trip, there are three altitudes that have two measurements (Laboratory at 150 m, Prunet at 250 m and Cammazes dam at 570 m). The purple dot is the value recorded by the CG-5 gravimeter at Puylaurens.

### 4.3. Altitude Variations (Airborne)

#### 4.3.1. Methodology of Drone Flight Survey

**Figure 11:**
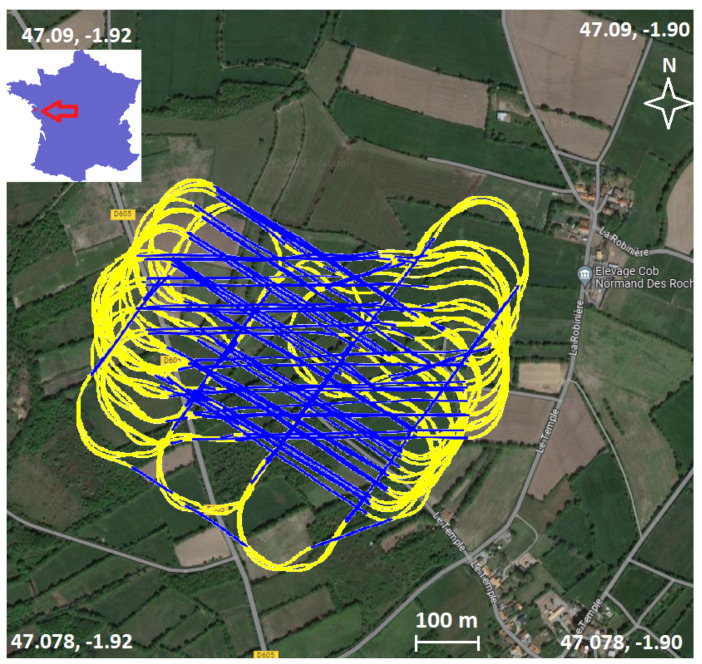
Map of flight area, with the complete flight trajectory of the drone (yellow + blue), the parts in blue are the straight parts.

The data used in this study were acquired over a period of four days from the 27 March until the 30 March 2021. The locations of the flights were in fields in the commune of Saint-Hilaire-de-Chaléons in Western France (Figure 11). During this period, these flights were conducted within the framework for another project [19].

The MEMS INS/GNSS data we used for this study, which are discussed later, were GNSS positioning (longitude, latitude and altitude) and accelerometric data (m/s²) for the *x*, *y* and *z*-axis, and also G (g), the velocity (m/s) of the UAV for all three axes, the temperature and time. All of which were recorded at a sampling frequency of 16 Hz.

The coordinates of the launch site were longitude: W 1°53${}^{\prime }$58.686${}^{\prime \prime }$ and latitude: N 47°5${}^{\prime }$21.6672${}^{\prime \prime }$. As mentioned before, each flight lasted for approximately 2 h, during which time the UAV flew on the same path. The reason for the UAV flying on the same paths instead of in circles was so that we had data from the UAV that were leveled horizontally, which greatly simplified the calculations and reduced the noise levels caused by the UAV’s movements. In order to produce data in different directions, the directions of the pathway was changed, and on top of that, the altitude was varied over the course of the flight. We obtained the Digital Elevation Model (DEM) from IGN, which allowed us to study the topography of this flight area. Surface topography does not exceed 20 m and the corresponding gravity effects due to topography changes (but not altitude changes) can be neglected. For altitude variations, theoretically, this corresponds to a change in acceleration of −0.3085 mGal/m × 20 ∼−6 mGal when going from a ground altitude H to an altitude of H + 20 m provided that the ground surface is flat and the ground under the UAV has a homogeneous density, which is, to first order, the case in our experiment.

By transforming the GNSS data recorded by the INS/GNSS from latitude (°), longitude (°) and altitude (m) to Cartesian coordinates (using the launch coordinates as the origin point), we obtain the coordinates of the flight in meters. By deriving these coordinates versus time, we can calculate the velocity along the x-, y- and z-axes, and by deriving this velocity dataset again, we can obtain the acceleration of the drone along its three axes. This information is used to reduce the signal of the acceleration recorded by the MEMS INS/GNSS by removing the part that is due to the movements of the drone and not due to gravity. This signal includes the noise of the previous signals, which comes from the MEMS INS/GNSS itself but also all the accelerations that are recorded because of the vibrations of the UAV, which happen because of the on-board instruments, the motor of the plane, the wind, etc. To eliminate even more noise that hides tiny gravity signals, we focus on straight lines, so that the UAV flies as horizontally as possible. However, even on a straight line, the drone has small roll, pitch and yaw variations, which are due to many phenomena: wind, acceleration changes of the drone, flight corrections or altitude changes. Because of this issue, acceleration orientations need to be corrected with a rotation matrix, using the pitch, roll and yaw recorded by the gyroscopes of the INS/GNSS.

**Figure 12:**
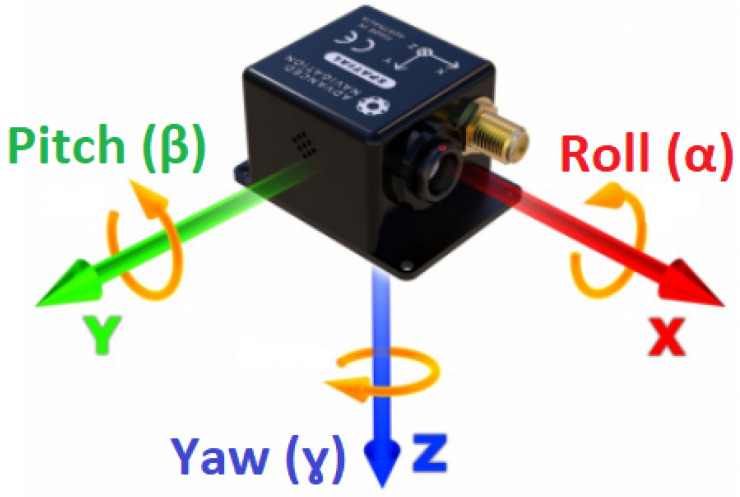
Schematic view of the SPATIAL model and the rotations; yaw or heading (γ), pitch (β) and roll (α) around the z-, y- and x-axes, respectively (from Advanced Navigation datasheet).

The first step to treating the flight data from a straight line is to multiply the velocity and acceleration measured by the INS with a rotation matrix [25] in order to have all vectors in the same quadrant. The rotation matrix R is obtained from the multiplication of the three matrices that describe the rotation along the x- ($R_{x}$), y- ($R_{y}$) and z-axis ($R_{z}$) for the angle of the roll (α), pitch (β) and yaw (γ) (see Figure 12). The rotation matrix is then multiplied with the column vector of the velocity and acceleration.

(3) $$\begin{array}{c} R=R_{x}\left(\alpha \right)R_{y}\left(\beta \right)R_{z}\left(\gamma \right)\;\\ =\left[\begin{array}{ccc} 1 & 0 & 0\\ 0 & cos\;\alpha & sin\;\alpha \\ 0 & -sin\;\alpha & cos\;\alpha \end{array}\right]\left[\begin{array}{ccc} cos\;\beta & 0 & -sin\;\beta \\ 0 & 1 & 0\\ sin\;\beta & 0 & cos\;\beta \end{array}\right]\left[\begin{array}{ccc} cos\;\gamma & sin\;\gamma & 0\\ -sin\;\gamma & cos\;\gamma & 0\\ 0 & 0 & 1 \end{array}\right]\;\\ =\left[\begin{array}{ccc} cos\;\beta \;cos\;\gamma & cos\;\beta \;sin\;\gamma & -sin\;\beta \\ sin\;\alpha \;sin\;\beta \;cos\;\gamma -cos\;\alpha \;sin\;\gamma & sin\;\alpha \;sin\;\beta \;sin\;\gamma +cos\;\alpha \;cos\;\gamma & sin\;\alpha \;cos\;\beta \\ cos\;\alpha \;sin\;\beta \;cos\;\gamma +sin\;\alpha \;sin\;\gamma & cos\;\alpha \;sin\;\beta \;sin\;\gamma -sin\;\alpha \;cos\;\gamma & cos\;\alpha \;cos\;\beta \end{array}\right]\;\\ R_{x}\left(\alpha \right)=\left[\begin{array}{ccc} 1 & 0 & 0\\ 0 & cos\;\alpha & sin\;\alpha \\ 0 & -sin\;\alpha & cos\;\alpha \end{array}\right]\;\\ R_{y}\left(\beta \right)=\left[\begin{array}{ccc} cos\;\beta & 0 & -sin\;\beta \\ 0 & 1 & 0\\ sin\;\beta & 0 & cos\;\beta \end{array}\right]\;\\ R_{z}\left(\gamma \right)=\left[\begin{array}{ccc} cos\;\gamma & sin\;\gamma & 0\\ -sin\;\gamma & cos\;\gamma & 0\\ 0 & 0 & 1 \end{array}\right] \end{array}$$

**Figure 13:**
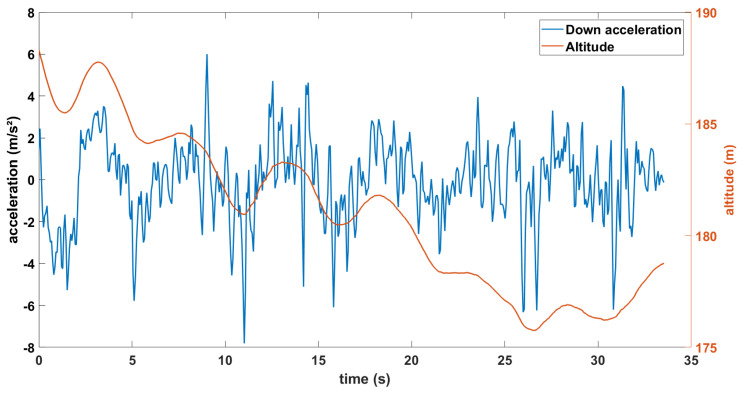
Altitude (red) and derived acceleration (blue), along the down axis of the drone during a straight line.

When looking at the data along a straight path, we can calculate the distance traveled along the *x*- and *y*-axis from the longitude and latitude data, and we can obtain the changes along the *z*-axis from the altitude data. With these positioning data and the sampling frequency of the MEMS INS/GNSS, we take the first derivative of this dataset versus time to obtain the velocity along these three axes, and then the second derivative to calculate the acceleration for each recording. However, deriving the time series generates numeric noise since the high frequencies are amplified, as well as edge effects. Multiple methods were tested to reduce the noise, but the best results were obtained by adding padded edges of zeros before and after the GNSS data, deconstructing the obtained signal down to its frequency domain, removing the amplitudes of the frequencies judged as noise (low-pass filter) and reconstructing the signal before deriving with a Lagrangian time derivative of degree 8. The MEMS INS/GNSS uses a mathematical algorithm for more precise recordings (up to 10 times more accurate than a traditional Kalman filter) in the case of a satellite dropout so this dataset contains little noise. Figure 13 presents the altitude data and the acceleration calculated from its second derivative.

We subtract the accelerometric data recorded by the INS with the calculated acceleration along the three axes. This allows us to remove the accelerometric signal from the INS data that is due to the movements of the drone.

The ellipsoid describes the mathematical surface that best represents the surface of the world’s oceans. The International Gravity Formula, including the height dependency, of the European Cooperation in Legal Metrology is described by Equation (4) [26,27], which is used mainly for European latitudes.

(4) $$\begin{array}{cc} g(\phi )= & 9.780327\left(1+0.0053024\sin^{2}\phi -0.0000058\sin^{2}2\phi \right)-3.085e10^{-6}h \end{array}$$

Using this formula, we can calculate the normal gravity for each point during the drone flight by inputting both the latitude (ϕ) and height (h). However, if we want a more accurate value of gravity, we need to include the effect of topography. To do so, we take the Digital Elevation Model (DEM) data for our flight zone to calculate the topography correction.

After removing the effect of the topography, which is very low as there are small to no topography changes for this (<5 m), the remaining gravity variations are mainly due to altitude variations. As seen in Equation (4), for every meter above the surface of the ellipsoid, there is a difference of 0.3085 mGal. Thus, our aim with the data from this experiment is to see the effect of the altitude variations on our accelerometric data.

#### 4.3.2. Results of Airborne Gravimetry Experiment

Because of the low topography changes and homogeneous nature of this region in terms of density, the data of the complete flight are examined instead of looking at just straight lines (which is what the other mentioned studies did). Another reason is because our straight lines only have a length of ∼1.2 km, and with an average velocity of 30 m/s, it would only take ∼30 s for each straight line. With a sampling frequency of 16 Hz, this would greatly limit our filtering capacity. As described in [9,16], their straight lines had a length of ∼100 km. With an average velocity of around 60 m/s, they would be filtered at 120 s, resulting in a spatial resolution of 3.6 km. With our flight area being around 2 km by 2 km, and our flight lasting 1 h, our drone flew over a distance of approximately 100 km as well. If we filter at 120 s over the total flight duration for an average velocity of 30 m/s, we obtain a spatial resolution of 1.8 km, approximately the length and width of our area.

First we looked at the start of the recorded data, which includes the accelerometric data along the down axis when the drone is being prepared, the motor is being turned on and the actual launch of the drone using a slingshot catapult. We use these startup data to look at the vibration effects that are due to the motor of the drone. We isolate the data between “Motor turned on” and “Launch”. Looking at the frequency spectrum, we remove the frequencies that are only due to the motor from the flight data. Because this part of the data was shorter than the complete flight, we interpolated it in order to subtract these data from the frequencies of the whole flight.

In a second step, we take the recorded value during the drone flight, after the drone has reached a stable altitude and before the landing procedure has started. As mentioned before, these accelerometric data are multiplied with the rotation matrix for each point so we are working in the NED (North, East, Down) frame, and we only consider the DOWN component of the acceleration, which corresponds to the vertical acceleration towards the mass center of the Earth. From this dataset, we remove the acceleration calculated by deriving the velocity given by the MEMS INS/GNSS (calculated from its GNSS coordinates) similarly to the process performed when analyzing the straight lines.

We notice there is still a considerate amount of noise remaining. We believed this noise came from the parts when the drone was turning so we compared this signal with that of the yaw because for every turn on the path, the yaw will change by approximately 180°, and we noticed the same amount of minimum and maximum values on both graphs.

Because gyroscopes drift over time, the amount of noise remaining could be due to the uncertainty of the angular values that were used for the rotation matrix.

**Figure 14:**
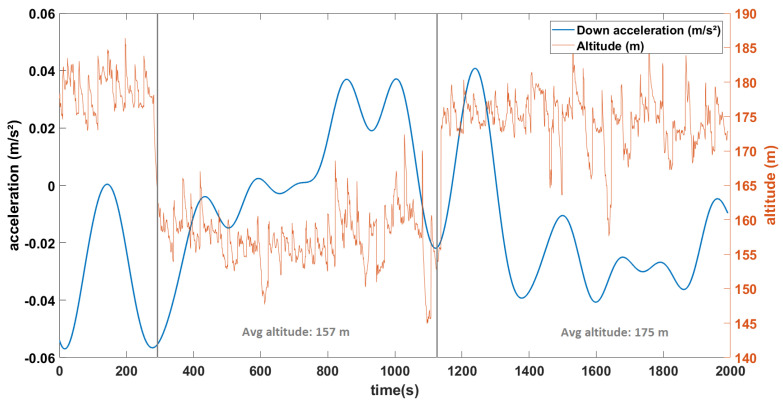
Down acceleration (m/s²) for the straight lines considered and the corresponding altitudes (m).

Instead of looking at the complete flight data, we now remove all turns. To do this, we filter out all points where the change in yaw between consecutive points or the roll values passes a threshold value (yaw > 1.0${}^{\circ }$ between two consecutive points or roll > 10${}^{\circ }$). In Figure 11, the data points that are kept are shown in blue. From here we proceed by using the same method as described earlier, using only the data corresponding to those points. Figure 14 shows the accelerometric data along the down axis following these steps, which shows more precise results.

## 5. Discussion of the Results for All Three Experiments

For our first experiment, we studied the influence of the temperature change on our recorded data. This step, often not mentioned or overlooked in many studies, proved to be crucial for our experiments as this MEMS INS/GNSS has a strong temperature bias. Using a linear fit, we obtained $g=6.210^{-3}T-10.192$ for the temperature range of 34–37.5 ${}^{\circ }$C, with an RMSE of 0.036 (m/s²). At higher (>45 ${}^{\circ }$C) or lower temperatures (<20 ${}^{\circ }$C), the recorded data do not always satisfy this linear law. Using these data, we corrected our in situ MEMS INS/GNSS and CG-5 data and compared them to the theoretical solid Earth tide signals. The XWT shows a high coherence for the periods of 12–24 h, which correspond to the fundamental periods ($M_{2}$, $S_{2}$ and $S_{1}$) associated with the solid Earth tides. This same correlation was obtained with the CG-5 data, but because the gravimeter had recorded uninterruptedly for a longer period of two months, there was also a correlation with the period corresponding to the fortnightly lunar cycle (14 days). The phase shift between the MEMS INS/GNSS and the theoretical signal, the black arrows in Figure 5, is close to zero at the beginning of the time series but increases during the time evolution until it reaches 90${}^{\circ }$ at the end of the experiment. The opposite phenomenon can be observed between CG5 and the model (Figure 6), which are 90${}^{\circ }$ out of phase at the beginning of the experiment and in phase at the end. Decomposing the MEMS INS/GNSS data into their frequency domain (FFT) reveals the frequencies corresponding to the periods around 12–24 h because their amplitude is much higher. By removing all the frequencies below 10 h and reconstructing the signal by IFFT, we obtained a signal close to the theoretical signal from Tsoft. We can still notice some dephasing, mostly in the middle part (07/09, Dep1) of the reconstructed signal. This is explained by the fact that we are using all the frequencies seen in Figure 7, which includes the frequencies where the INS/GNSS data have a higher amplitude than Tsoft. These frequencies correspond to other signals (part of them being noise) recorded during the experiment and not a priori from the solid Earth tides. An examples of the multiple possible origins of these signals is the AC that turned on, as this experiment took place during the summer, with human activity around. The other difference is the amplitude between both signals for the middle (07/09, Dep1) and last part (11/09, Dep2). When we compared this to the atmospheric pressure recorded, we noticed a decrease in the pressure for those same periods. Since this is not included in the Tsoft theoretical model, we suspect that decreases in atmospheric pressure result in a lower load on the continental crust, which decreases the gravity amplitude change of its deformation.

For our experiment at the Cammazes dam, to see if the MEMS INS/GNSS was able to sense the gravity changes due to the altitude variations, our previously established thermal drift correction is crucial again. The values obtained before temperature correction (red crosses) were far off from the reference values of the CG-5 (blue circles). After temperature correction, $g_{d}$ (green dots), the data are much closer to the reference points in Figure 10. The RMSE between the INS/GNSS and the CG-5 (our reference) is $3\times 10^{-5}$ (m/s²). At the three locations that we looped back to, with the GET laboratory being the starting and the end point (150 m altitude), we notice that the INS/GNSS data drifts further away from the CG-5 the longer the mission lasts because of the increased inaccuracy due to the temperature differences. Nonetheless, the final result (green dots) shows that the MEMS INS/GNSS did sense the gravity variations due to the altitude considering that the latitude barely changed.

**Figure 15:**
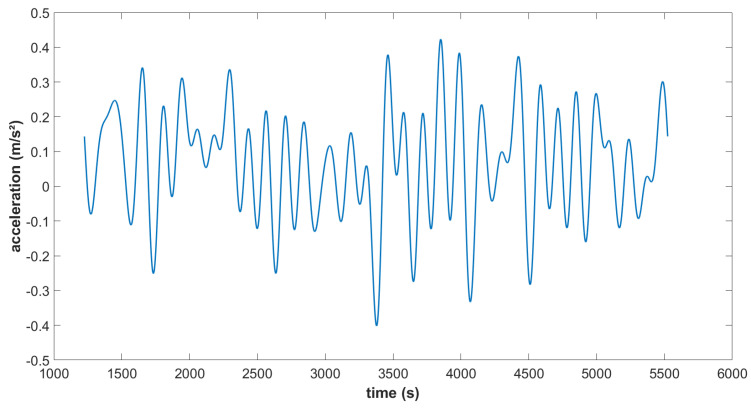
Down acceleration for the complete flight, after corrections and filtering.

For the drone experiment, the MEMS INS/GNSS was installed in a fixed-wing rotor drone. Multiple methods for analysis were tested and we ended with the configuration that was the best balance between keeping as much data as possible, while also removing as many of the factors that add noise or signals that are not of interest (drone turning, motor vibrations, wind, INS/GNSS measurement noise, etc.). The best result, which was obtained after a temperature correction, and after applying the rotation matrix and removing the noise caused by the motor vibrations, the kinematic accelerations and all the parts of the trajectory that correspond to the drone turning, is seen in Figure 14. Compared to Figure 15 which presents the complete trajectory, we obtain a result that fluctuates less in both frequency and amplitude. Although the turns were eliminated with a filter that uses the roll and yaw, there are still changes in pitch on straight lines (rotation along the *y*-axis). Any drift in gyroscopes, which also affect the recordings of the pitch and cannot be corrected, therefore affect our remaining accelerometric signal. The difference in the average value between the second part (average altitude 157 m) and third part (average altitude 175 m) is 31 mGal, which is 6 times higher than the calculated theoretical value of 5.5 mGal for this altitude variation (altitude calculated from the ellipsoid model since topography changes are negligible in this area). From the Cammazes dam experiment, we demonstrated that this instrument can obtain a higher precision (<5 mGal) when it comes to gravity changes due to altitude variations, but that precision was not obtained here because of the many different vibrations on the drone. In the final chapter, we discuss possible improvements for future studies in order to remove more noise.

## 6. Conclusions and Perspectives

Gravimeters are usually used to study the Earth’s gravity field variations but they remain heavy and expensive, whereas an INS/GNSS MEMS unit provides accelerometric data to correct navigation data that can be used opportunistically to measure gravity since gravity is considered an acceleration. INSs/GNSSs have already been used in mobile gravimetry experiments [1,16], but this is the first time that such a light, relatively low-cost and easy to install device has been used. The MEMS gravimeters that have been used to study the solid tides [12,13] are definitely more portable than a commercial gravimeter, attaining a 91% and 97.5% correlation, respectively. However, even with their reduced size and weight, they still cannot be used for mobile experiments (for example, our Cammazes dam experiment) because the MEMS gravimeter needed to be placed inside a vacuum chamber on top of a marble table to ensure it was placed perfectly horizontal.

Using this type of MEMS INS/GNSS for in situ experiments to detect the signal of solid Earth tides has not been done before. While the data recorded by this miniature MEMS INS/GNSS are very noisy due to various factors (the MEMS INS/GNSS error, motor residual vibrations, wind and turbulence effects, thermal drift, etc.), after recording enough data in static mode and applying the empirical corrections to remove the thermal drift, the 10 h low-pass-filtered accelerometric data reveal the typical 12 and 24 h tidal oscillations that represent 90–95% of the values simulated by the Tsoft software [21]. Higher amplitudes of the tidal oscillations found by accelerometry may be caused by noise due to external vibrations because they do not affect the high frequencies. When the measured INS/GNSS signal is low-pass filtered at sub-10 h frequencies, we obtain a signal close to the theoretical one obtained from Tsoft with a correlation of 0.84. The difference between the INS-measured and Tsoft values seems to be correlated to atmospheric pressure, and the gap is particularly important during low-pressure (Dep1 and Dep2) periods. We attained sufficient precision to obtain the acceleration signal caused by the solid Earth tides (<1 mGal).

Similarly, during an experiment conducted from the GET laboratory to the Cammazes dam, we were able with this MEMS INS/GNSS to attain a high enough precision to present the influence of the altitude variations on the gravitational acceleration (<5 mGal).

The data recorded when the MEMS INS/GNSS is embedded in a drone, installed close to the drone’s center of mass, showed promising results. The velocity and GNSS data, which are calculated using an algorithm developed by Advanced Navigation that is up to 10 times more accurate than a traditional Kalman filter, are precise enough to derive the acceleration components. Through high-frequency sampling of accelerations, velocity, GNSS information, pitch, roll, yaw, angular velocity, etc., at high frequencies, we can effectively use this dataset to reduce the accelerometric signal recorded during the flight. We filtered this reduced signal at 0.0083 Hz (or 120 s) because with an average velocity of 30 m/s, this gives us a spatial resolution of 1.8 km, the approximate length and width of our flight area, which has both a homogeneous soil density and no significant topography variations. The remaining signal after all corrections should be equal to that of the drone at rest with the motor turned off and only presenting the altitude’s influence. By changing its altitude, the drone sensed the change in gravity, conformal to the upward (or downward) continuation of 0.3085 mGal/m, but hardly detectable because of the presence of noise of a higher order of magnitude.

Previous airborne gravimetry studies have provided a <5 mGal precision when flying over the same straight (100 km) lines with a spatial resolution of 4.8 km [1,16]. Although our accelerometer data suffer from very important noise reaching +/− 1 g, the system in static mode is still able to recover sub-mGal variations in geophysical signals such as the main solid tides as well as pure altitude changes (see the Cammazes dam experiment). Our precision (25 mGal) during airborne gravimetry can definitely be improved upon if we want to achieve the desired precision for gravimetric applications (<5 mGal).

More studies should be performed using MEMS INS/GNSS for in situ experiments as these experiments showed the best results. Different locations could be tested, such as a cave where there is little to no vibrations caused by the presence of anthropogenic noise and the temperature is quite constant (annual temperature range 12–14 ${}^{\circ }$C). This system could also be used for an in situ experiment to detect the gravity variations due to the presence of an aquifer.

For future airborne experiments, while the duration of the flight (∼1–1.5 h) is perfect for this type of instrument, the temperature bias and natural drift of the MEMS INS/GNSS remain too high.

As well as the thermal effects and instrumental drift of the MEMS, longer drone trajectories formed by straight lines at a constant altitude would ensure easier-to-interpret records of acceleration in the future. A longer study should also be conducted with the drone at rest and the motor turned on to have longer records of the accelerations due to the motor. The MEMS INS/GNSS should also be attached to a better shock-absorbing material to damp the mechanical vibrations. While it will be impossible to remove all vibrations due to the motor, the more high-amplitude/high-frequency signals that are removed by well-adapted filtering, the better the isolation of the gravity component will be. The MEMS INS/GNSS could also be fixed on a gimbal so that the data of the gyroscopes (which naturally drifts) do not need to be used for the rotation matrix in order to work in the NED-frame.

## Figures and Tables

**Table 1:** Navigation specifications.

Parameter	Value
Horizontal position accuracy (with L1 RTK)	0.02 m
Vertical position accuracy (with L1 RTK)	0.03 m
Velocity accuracy	0.05 m/s
Roll and pitch accuracy (dynamic)	0.2°
Heading accuracy (dynamic with GNSS)	0.2°

**Table 2:** Sensor specifications.

Parameter	Accelerometers	Gyroscopes
Bias instability	20 µ g	3${}^{\circ }$/h
Initial bias	<5 mg	<0.2${}^{\circ }$/s
Scale factor stability	<0.06%	<0.05%
Noise density	100 µg/$\sqrt{Hz}$	0.004°/s/$\sqrt{Hz}$

**Table 3:** CG-5 Scintrex specifications.

Embedded Sensor	Fused Quartz w/ Electrostatic Nulling
Resolution	1 µGal
σ	<5 µGals
Operating range	8000 mGal
Residual long term static drift	<0.02 mGal/day
Range of tilt compensation	±200 arc sec
Corrections	Tides, instrument tilt, temp
GPS accuracy	With WAAS correction < 3 m
Battery properties	2 × 6.6 Ah (11.1 V) Li batteries
Power consumption	6.5 Watt at 25 ${}^{\circ }$C
Operating temperature	−40 ${}^{\circ }$C to +55 ${}^{\circ }$C
Output	USB memory stick, RS-232C

## Data Availability

The data presented in this study are available on request from the corresponding author.

## Abbreviations

The following abbreviations are used in this manuscript:

INS	Inertial navigation system
SINS	Strapdown inertial navigation system
GNSS	Global navigation satellite system
MEMS	Microelectronic mechanical systems
UAV	Unmanned aerial vehicle
GPS	Global Positioning System
GET	Géosciences Environnement Toulouse
FFT	Fast Fourier Transform
IFFT	Inverse Fast Fourier Transform
RMSE	Root-Mean-Square error
GRACE	Gravity Recovery and Climate Experiment
CHAMP	Challenging Minisatellite Payload
GOCE	Gravity Field and Steady-State Ocean Circulation Explorer

## References

[1] Lin C., Chiang K., Kuo C. (2017). Development of INS/GNSS UA-Borne Vector Gravimetry System. IEEE Geosci. Remote Sens. Lett..

[2] Gerlach C., Dorobantu R. A Testbed for Airborne Inertial Geodesy: Terrestrial Gravimetry Experiment by INS/GPS. Proceedings od the CD, IAG Symposium on Gravity, Geoid and Space Missions (GGSM 2004).

[3] Featherstone W.E. (2003). Improvement to long-wavelength Australian gravity anomalies expected from the CHAMP, GRACE and GOCE dedicated satellite gravimetry missions. Explor. Geophys..

[4] Flechtner F., Reigber C., Rummerl R., Balmino G. (2021). Satellite Gravimetry: A review of its realization. Surv. Geophys..

[5] Harriet C.P., Lau S.M. (2023). A Journey through Tides, Chapter 15 Solid Earth Tides.

[6] Jekeli C. (2001). Inertial Navigation Systems with Geodetic Applications.

[7] Kwon J.H., Jekeli C. (2000). A new approach for airborne vector gravimetry using GPS/INS. J. Geod..

[8] Zhang K.D. (2007). Research on the Methods of Airborne Gravimetry Based on SINS/DGPS.

[9] Cai S., Wu M., Zhang K., Cao J., Tuo Z., Huang Y. (2013). The first airborne scalar gravimetry system based on SINS/DGPS in China. Sci. China Earth Sci..

[10] Senobari M.S. (2010). New results in airborne vector gravimetry using strapdown INS/DGPS. J. Geod..

[11] Xia Z., Sun Z.M. (2006). The technology and application of airborne gravimetry. Sci. Surv. Mapp..

[12] Tang S., Liu H., Yan S., Xu X., Wu W., Fan J., Tu L. (2019). A high-sensitivity MEMS gravimeter with a large dynamic range. Microsystems Nanoeng..

[13] Prasad A., Middlemiss R.P., Noack A., Anastasiou K., Bramsiepe S.G., Toland K., Utting P.R., Paul D.J., Hammond G.D. (2022). A 19 day earth tide measurement with a MEMS gravimeter. Sci. Rep..

[14] De Saint Jean B. (2008). Étude et Développement d’un Système de Gravimétrie Mmobile. Ph.D. Thesis.

[15] Verdun J., Roussel C., Cali J., Maia M., D’Eu J.F., Kharbou O., Poitou C., Ammann J., Durand F., Bouhier M.E. (2022). Development of a Lightweight Inertial Gravimeter for Use on Board an Autonomous Underwater Vehicle: Measurement Principle, System Design and Sea Trial Mission. Remote Sens..

[16] Luo K., Cao J., Wang C., Cai S., Yu R., Wu M., Yang B., Xiang W. (2022). First unmanned aerial vehicle airborne gravimetry based on the CH-4 UAV in China. J. Appl. Geophys..

[17] Cai S.K., Zhang K.D., Wu M.P., Cao J.L. (2015). Airborne Vector Gravimetry based on SINS/DGPS. Hydrogr. Surv. Charting.

[18] Cai S.K., Zhang K.D., Wu M.P. (2015). Study on Airborne Gravity Vector Measurement and Error Separation Method.

[19] Alaoui-Sosse S., Durand P., Médina P. (2022). In Situ Observations of Wind Turbines Wakes with Unmanned Aerial Vehicle BOREAL within the MOMEMTA Project. Atmosphere.

[20] Alaoui-Sosse S., Durand P., Medina P., Pastor P., Gavart M., Pizziol S. (2022). Boreal, a Fixed-Wing Unmanned Aerial System for the Measurement of Wind and Turbulence in the Atmospheric Boundary Layer. J. Atmos. Ocean. Technol..

[21] Van Camp M., Vauterin P. (2005). Tsoft: Graphical and interactive software for the analysis of time series and Earth tides. Comput. Geosci..

[22] Na S.-H. (2021). Prediction of Earth tide. Basics of Computational Geophysics.

[23] Grinsted A., Moore J.C., Jevrejeva S. (2004). Application of the cross wavelet transform and wavelet coherence to geophysical time series. Nonlinear Process. Geophys..

[24] Gaillot P., Darrozes J., de Saint Blanquat M., Ouillon G. (1997). The normalised anisotropic wavelet coefficient (NOAWC) Method: An image processing tool for multi-scale analysis of rock fabric. Geophys. Res. Lett..

[25] Slabaugh G.G. (2000). Computing Euler angles from a rotation matrix. Retrieved August.

[26] Lambert W.D. (1945). The International Gravity Formula.

[27] Schwartz R., Lindau A. (2003). Das europaische Gravitationszonenkonzept nach WELMEC für eichpflichtige Waagen. PTB-Mitteilungen.

